# Syntheses and Cell-Based Phenotypic Screen of Novel 7-Amino pyrido[2,3-*d*]pyrimidine-6-carbonitrile Derivatives as Potential Antiproliferative Agents

**DOI:** 10.3390/molecules17032351

**Published:** 2012-02-24

**Authors:** Tao Yang, Hong He, Wei Ang, Ying-Hong Yang, Jian-Zhong Yang, Yan-Ni Lin, Hua-Cheng Yang, Wei-Yi Pi, Zi-Cheng Li, Ying-Lan Zhao, You-Fu Luo, Yuquan Wei

**Affiliations:** 1 State Key Laboratory of Biotherapy, West China Hospital, West China Medical School, Sichuan University, Chengdu, Sichuan 610041, China; 2 Key Laboratory of Drug Targeting and Drug Delivery System, Ministry of Education, West China Medical School, Sichuan University, Chengdu, Sichuan 610041, China; 3 Department of Pharmaceutical and Bioengineering, School of Chemical Engineering, Sichuan University, Chengdu, Sichuan 610065, China

**Keywords:** 7-aminopyrido[2,3-*d*]pyrimidin-6-carbonitrile derivatives, cell-based phenotypic screening, anti-tumor activity, structure-activity relationship (SAR), apoptosis

## Abstract

A series of N-3-substituted 7-aminopyrido[2,3-*d*]pyrimidin-6-carbonitrile derivatives was readily synthesized and their anti-proliferative activities on five types of tumor cells were evaluated through a cell-based phenotypic screening approach. Compound **3k** was found to be potent on human colon cancer SW620 cells with an IC_50_ value of 12.5 μM. Structural optimization of compound **3k** led to compound **4a** with improved anti-proliferative potency on SW620 cells with an IC_50_ value of 6.9 μM. Further cell-cycle analyses suggested that compound **4a** induced apoptosis of SW620 cells in a concentration-dependent manner.

## 1. Introduction

Chemotherapy is one of the most commonly used treatment options for malignant tumors, especially for unresectable patients [1]. Improvements in treatment and prevention have led to a decrease in cancer deaths, but the number of new diagnoses continues to rise. New classes of therapies targeting specific proteins perturbed in cancers have been heralded as “smart drugs” that more effectively target the disease than current chemotherapeutic regimes such as doxorubicin, cisplatin and fluorouracil [2]. However, disappointing results in recent clinical trials indicate that a major challenge of target-based drug discovery approaches is overcoming target mechanism heterogeneity among patients and inherent or acquired drug resistance [3]. Consequently, current cancer drug discovery approaches are not appropriately tailored to complex disease mechanism(s) [4]. Phenotypic screening, widely used to find new drugs in old days, is arguably associated with the more humble recognition that you really don’t believe you understand the mechanism [5]. If you can establish a way to make a wayward cell less harmful, the mechanism may not matter all that much. Between 1999 and 2008, the contribution of phenotypic screening to the discovery of first-in-class small-molecule drugs exceeded that of target-based approaches-with 28 and 17 of these drugs coming from the two approaches, respectively [5].

Our research group focused our attention on the design, synthesis and cell-based phenotypic screening of novel tumor growth inhibitors and apoptosis inducer as potential anti-proliferative agents. In recent years nitrogen-containing heteroaromatic species of biological significance have attracted considerable research interest as these entities constitute the core structure of numerous pharmaceuticals. Pyrimidine derivatives have shown remarkable activity as PDE4 inhibitors, antileukemia, bronchodilators, vasodilators, antiallergic, antihypertensive and anticancer agents [6,7,8,9,10,11,12]. In the past few years, several synthetic methodologies for 7-aminopyrido[2,3-*d*]pyrimidin-6-carbonitrile derivatives have been developed [13]. However, the antitumor activities of these compounds have not been fully explored.

**Figure 1:**
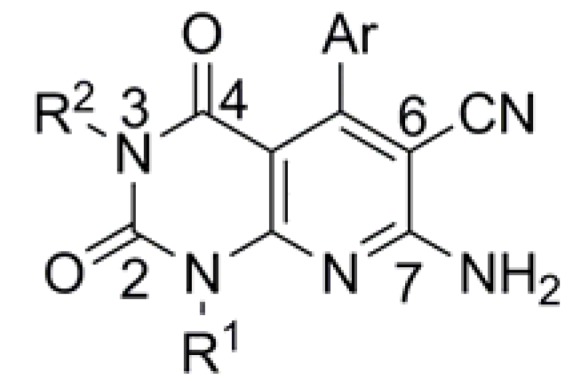
Structure of 7-amino-pyrido[2,3-*d*]pyrimidine-6-carbonitrile derivatives.

In this article we describe a novel series of N-3-substituted 7-aminopyrido[2,3-*d*]pyrimidine-6-carbonitrile derivatives (Figure 1) and a cell-based phenotypic evaluation of their anti-tumor activities by the MTT method. The cell-cycle analysis of the most potent compound **4a** is presented. Their preliminary structure–activity relationships (SARs) are also discussed. 

## 2. Results and Discussion

### 2.1. Chemistry

**Scheme 1:**
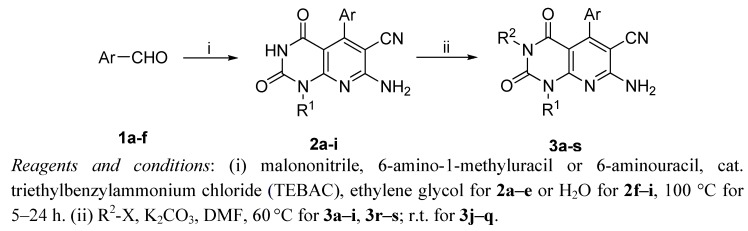
Synthetic route of compounds **3a–s**.

The general synthetic approach to the desired final compounds **3a**–**s** is outlined in Scheme 1. 

The core structure of 7-aminopyrido[2,3-*d*]pyrimidine-6-carbonitrile derivatives, was built *via* a reported multi-component reaction [14] with minor revisions. Briefly, the key intermediates **2a–i** were readily synthesized in parallel with an appropriate aromatic aldehyde, 6-methyl-1-amino uracil/6-aminouracil and malononitrile as starting materials in the presence of TEBAC. The final compounds **3a–s** were thus obtained by N-3 alkylation of the precursors **2** with R^2^-X in the presence of potassium carbonate in DMF. To note, derivatives **3a–i** and **3r–s** were obtained at 60 °C, whereas the reaction at room temperature was found to be optimal to afford compounds **3j–q**. The structures of **3a–s** were fully characterized and identified by ^1^H-NMR, ^13^C-NMR and HR-MS analyses.

**Scheme 2:**
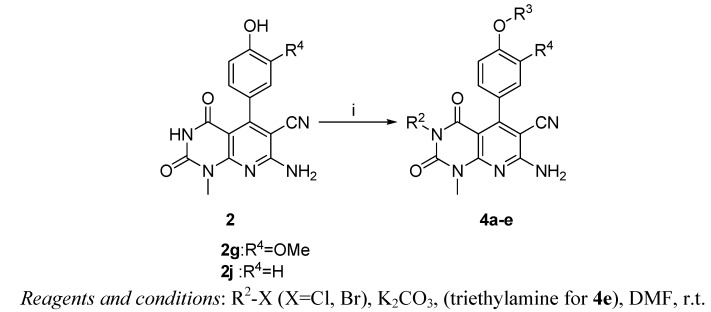
Synthetic route of compounds **4a–e**.

As illustrated in Scheme 2, to find a more potent compound, derivatives **4a–e** were prepared starting from **2g** or **2j** in the presence of K_2_CO_3_ or triethylamine at room temperature in DMF. Compounds **4a–e** were fully characterized and identified by ^1^H-NMR, ^13^C-NMR and HR-MS before biological evaluation.

### 2.2. Anti-proliferative Activities

All the final compounds underwent primary phenotypic screening for their inhibitory activity against A549, HepG2, SW620, Skov-3 and HeLa tumor cells at 40 μM using the MTT assay. For comparison, the data of cisplatin are also included. As shown in Table 1, some derivatives (compounds **3j–s**) exerted potent or moderate inhibitory activities against the five assayed tumor cell lines at 40 μM (Table 1). The inhibitory activities of most compounds on SW620 cells were slightly more potent than those for human lung carcinoma A549 cells, human hepatocellular liver carcinoma HepG2 cells, human cervical carcinoma epithelial HeLa cells and human ovarian cancer Skov-3 cells. In our initial efforts to search for novel potent anticancer agents, a caffeine moiety, reported to suppress the proliferation of various cancer cell lines and transformed cell lines [15,16], was covalently coupled to the 7-aminopyrido[2,3-*d*]pyrimidine-6-carbonitrile scaffold through a two-carbon linker at the N-3 position. Unfortunately, the obtained compound **3a** demonstrated low inhibitory effects on the five tested tumor cell lines. Further modification of compound **3a** with various substituents at the C-5 position of the pyrido[2,3-*d*]pyrimidine scaffold showed no improvement in activities. 

**Table 1:** Inhibition of compounds **3a–s** on five types of tumor cells at 40 μM. 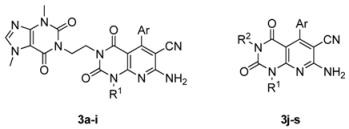

Compd.	R^1^	R^2^	Ar	IN ^a^ at 40 μM (%)				
				SW620	A549	SKOV-3	HepG2	HeLa
3a	H	—	Phenyl	9 ± 2.3	10 ± 2.6	NT ^b^	NT	37 ± 3.0
3b	H	—	4-Cl-Ph-	8 ± 1.4	3 ± 1.5	12 ± 2.2	NT	18 ± 2.1
3c	H	—	3,4-di MeO-Ph-	11 ± 0.9	22 ± 0.5	NT	29 ± 2.8	29 ± 2.1
3d	H	—	3-MeO-4-OH-Ph-	13 ± 3.0	9 ± 2.2	18 ± 2.4	10 ± 1.8	20 ± 1.1
3e	H	—	4-Br-Ph-	27 ± 1.2	28 ± 2.9	NT	21 ± 2.8	34 ± 1.6
3f	Me	—	Phenyl	7 ± 1.7	NT	NT	NT	NT
3g	Me	—	3-MeO-4-OH-Ph-	3 ± 1.3	NT	NT	NT	17 ± 3.1
3h	Me	—	3,4-di MeO-Ph-	10 ± 0.8	16 ± 1.7	NT	17 ± 2.2	23 ± 0.7
3i	Me	—	Thiazolyl	NT	NT	NT	NT	NT
3j	Me	2-methylbenzyl	3,4-di MeO-Ph-	14 ± 2.1	NT	NT	NT	NT
3k	Me	2-methylbenzyl	3-MeO-4-(2-Me-BnO)-Ph	85 ± 2.8	26 ± 3.6	NT	28 ± 3.3	9 ± 4.0
3l	Me	2-methylbenzyl	Phenyl	36 ± 0.9	NT	NT	10 ± 3.8	NT
3m	Me	2-fluorobenzyl	Phenyl	48 ± 4.3	52 ± 2.3	32 ± 2.1	20 ± 1.9	NT
3n	Me	3-fluorobenzyl	Phenyl	42 ± 3.1	55 ± 1.1	NT	16 ± 0.5	14 ± 2.8
3o	Me	2-(4-fluorophenyl)-2-oxoethyl	Phenyl	38 ± 1.7	22 ± 3.9	8 ± 2.5	24 ± 5.4	23 ± 1.9
3p	Me	2-(4-methoxyphenyl)-2-oxoethyl	Phenyl	21 ± 0.9	38 ± 2.8	38 ± 1.0	20 ± 1.4	NT
3q	Me	Propargyl	Phenyl	25 ± 3.7	28 ± 1.6	7 ± 2.9	20 ± 3.3	19 ± 0.8
3r	Me	Cyclopentyl	Phenyl	52 ± 2.2	NT	NT	21 ± 1.7	14 ± 3.6
3s	Me	Butyl	Phenyl	35 ± 1.5	20 ± 1.8	NT	NT	13 ± 4.5
Cis ^c^	—	—	—	72 ± 2.1	68 ± 1.8	67 ± 1.9	80 ± 2.8	80 ± 3.5

^a ^IN = inhibition, measured 48 h after treatment with compounds **3a–s** (40 μM). Results are given in concentrations of 40 μM after continuous exposure of 48 h and show means ± SEM values of three-independent experiments. IR = 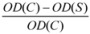; ^b ^NT denotes not tested; ^c ^Cis denotes cisplatin.

This finding could have been due to the excessive numbers of hydrophilic nitrogen atoms in the caffeine substituent. Hence, coupling caffeine with the pyrido[2,3-*d*]pyrimidine scaffold did not work as expected. Thus, we turned to link more lipophilic groups at N-3 position to investigate whether thus obtained derivatives would inhibit tumor cell proliferation more effectively. Compounds **3j–s**, with certain lipophilic alkyl or aryl groups incorporated into the N-3 position, exhibited higher inhibition values (Table 1) as proposed.

To obtain more accurate data on the anti-proliferative activities of derivatives **3j–s**, we conducted an MTT assay against SW620 tumor cells to measure their IC_50_ values. The concentrations of the assayed compound are in a range from 2.5 to 80 μM. As shown in Table 2, derivative **3k** was found to be the most potent one against human colon cancer cells SW620 at low micromolar level, with an IC_50_ value of 12.5 μM, comparable to that of cisplatin. However, compound **3k** exhibited extremely poor inhibitory potency for A549, Skov-3, HepG-2 and HeLa cell lines (Table 1). 

**Table 2:** IC_50_ values of compounds **3j–s** on SW620 cells.

Compd.	IC_50_^a^(μM)	Compd.	IC_50_(μM)	Compd.	IC_50_(μM)	Compd.	IC_50_(μM)
**3j**	>80	**3p**	59.6 ± 2.1	**3m**	65.1 ± 1.1	**3s**	>80
**3k**	12.5 ± 0.7	**3q**	71.9 ± 1.5	**3n**	79.6 ± 2.7	Cis ^b^	9.5 ± 0.5 ^c^
**3l**	76.1 ± 1.9	**3r**	29 ± 0.9	**3o**	71.5 ± 1.8		

^a ^IC_50_ denotes half maximal inhibitory concentration. Values are means ± SEM of three independent experiments; ^b ^Cis denotes cisplatin; ^c^ The IC_50_ value is comparable to the reported in the literature [17].

To find a more potent compound, derivatives **4a–e** were readily prepared starting from **2g** or **2j** in the presence of K_2_CO_3_ or triethylamine at room temperature in DMF (Scheme 2). Compounds **4a–e** were fully characterized and identified by ^1^H-NMR, ^13^C-NMR and HR-MS before biological evaluation. The IC_50_ values of compounds **4a–e** on SW620 tumor cells are shown in Table 3.

**Table 3:** IC_50_ values of compounds **4a–e** on SW620 cells. 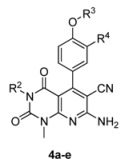

Compd.	R^2^	R^3^	R^4^	IC_50_^a^(μM)
**4a**	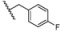	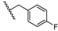	OMe	6.9 ± 0.4
**4b**	“	“	H	>40
**4c**	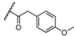	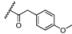	OMe	>40
**4d**	“	“	H	>40
**4e**	H	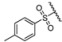	OMe	36.8 ± 1.1
**Cis ^b^**	-	-	-	9.5 ± 0.5

^a ^IC_50_ denotes half maximal inhibitory concentration. Values are means ± SEM of three independent experiments; ^b ^Cis denotes cisplatin.

Special emphasis was placed upon SAR studies on the 3-position of the pyrido[2,3-*d*]pyrimidine scaffold and 3-, 4-positions of the phenyl(Ar) at the 5-position of the scaffold (Table 3). When R^4^ was a methoxy group, R^2^ and R^3^ were substituted by the benzyl or 1-phenylethanone-2-yl group, respectively, and two compounds, **4a** and **4c**, were obtained with notably different IC_50_ values on SW620 cells, 6.9 μM for **4a** and above 40 μM for **4c**. This result suggested that the carbonyl group of the 1-phenylethanone-2-yl substituent decreased anti-proliferative activities. We next investigated if the methoxy group (R^4^) at C-5 position of the pyrido[2,3-*d*]pyrimidine scaffold was necessary by removing the methoxy group. Consequently, compound **4b** was synthesized with a hydrogen atom for R^4^, which led to almost total loss of activity (Table 3). Thus, a methoxy group of R^4^ plays a crucial part in contributing to biological activities for pyrido[2,3-*d*]pyrimidine derivatives. 

### 2.3. Cell-Cycle Analyses

Cell-cycle analyses were done by flow cytometric measurements on SW620 cells. annexin V-fluorescein isothiocyanate (FITC) was used as a marker of phosphatidylserine exposure and propidium iodide (PI) as a marker for dead cells. This combination allowed differentiation between early apoptotic cells (annexin V-positive, PI-negative), late apoptotic/necrotic cells (annexin V-positive, PI-positive), and viable cells (annexin V-negative, PI-negative). 

**Figure 2:**
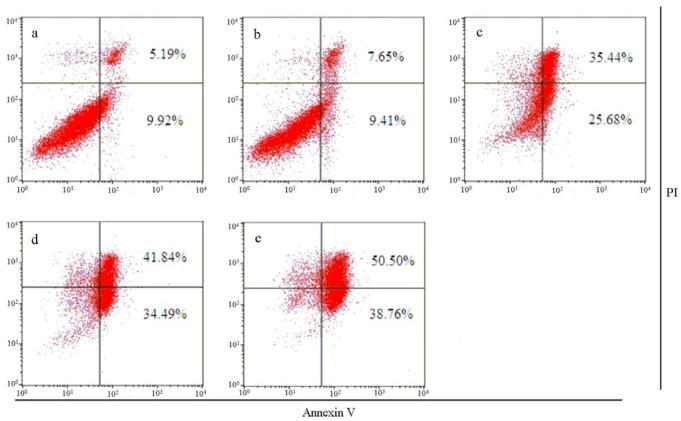
Effects of **4a** on the induction of apoptosis in SW620 cells for 48 h: a, control; b, 5 μM; c, 10 μM; d, 20 μM; e, 40 μM. Cells were stained with annexin V-FITC and PI. The total number of apoptotic cells are the sum of annexin V+/PI− (early apoptotic) and annexin V+/PI+ (late apoptotic/necrotic) cell populations.

After treatment with compound **4a** at 0, 5, 10, 20 and 40 μM for 48 h, the percentage of apoptotic cells was 15.11%, 17.06%, 61.12%, 76.33% and 89.26% (Figure 2). These results indicated that the growth inhibition of SW620 cells was caused by inducing apoptosis in a concentration-dependent manner [18]. 

## 3. Experimental

### 3.1. General

Human cancer cell lines were purchased from American Type Culture Collection (ATCC, Rockville, MD, USA). Dulbecco’s modified Eagle’s medium (DMEM) and RPMI 1640 were purchased from Gibco (Grand Island, New York, NY, USA). Fetal bovine serum (FBS) was purchased from Hyclone (Logan, UT, USA). All chemicals were commercially available and used without further purification unless otherwise stated. Column chromatography was carried out on silica gel (400 mesh, Qingdao Marine Chemical Ltd., Qingdao, China). Thin-layer chromatography (TLC) was undertaken on TLC silica gel 60 F254 plates. ^1^H-NMR and ^13^C-NMR spectra were recorded on a Bruker Avance (Varian Unity Inova) 400 MHz spectrometer using TMS as internal reference chemical shift in δ, ppm. Chemical shifts (δ) are reported in parts per million relative to tetramethylsilane (TMS) used as an internal standard, where (δ) TMS = 0.00 ppm. High-resolution mass spectrometry was carried out on a Waters Q-TOF Premier mass spectrometer.

### 3.2. General Procedure for the Synthesis of ***2a–j***

**Figure 3:**
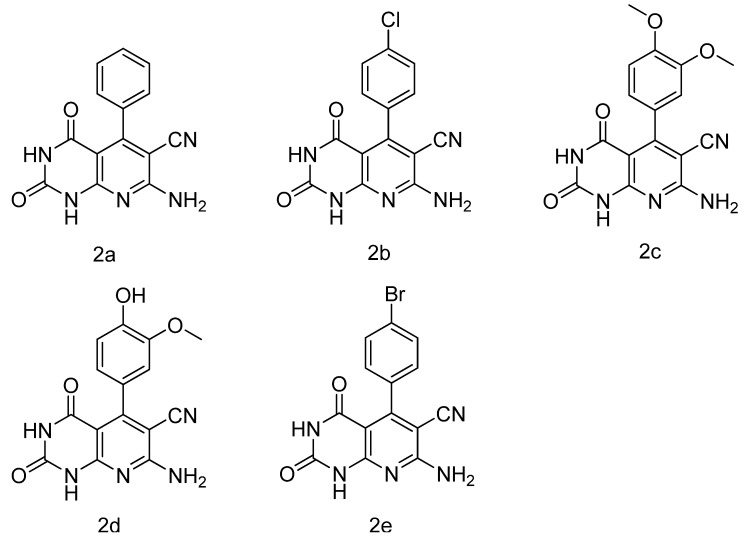
The chemical structure of compounds **2a–e**.

For derivatives **2a–e** (Figure 3), 6-aminouracil (10 mmol) were suspended in ethylene glycol (120 mL) at 100 °C, the appropriate aldehyde (10 mmol) and propanedinitrile (10 mmol) were added in one portion. 

After the mixture turned transparent, TEBAC (150 mg) was added to the mixture as phase transfer catalyst. The resultant mixture was stirred at 100 °C for 4–7 h, cooled to room temperature, The mixture was filtered, washed with EtOH, the solids **2a–e** formed were recrystallized from DMF and water. Their physical appearance, melting point and spectroscopic data were in agreement with published data.

**Figure 4:**
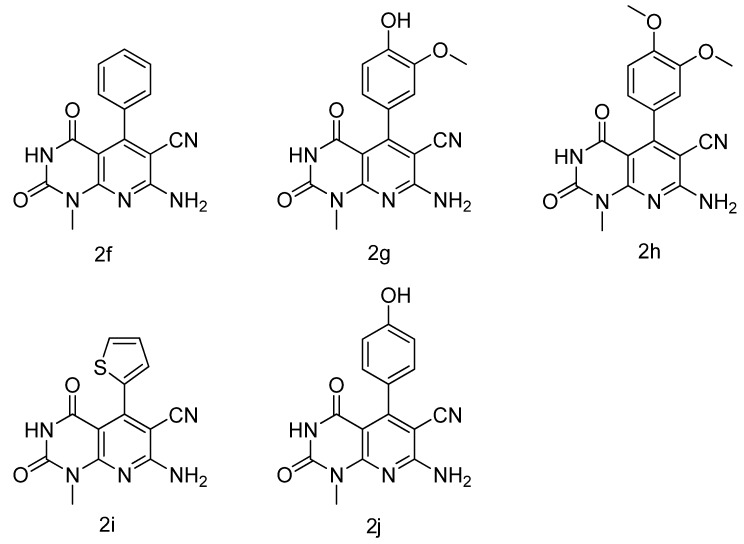
The chemical structure of compounds **2f–j**.

For derivatives **2f–j** (Figure 4), 6-amino-1-methyluracil (10 mmol) were suspended in water (120 mL) at 100 °C, aldehyde (10 mmol) and propanedinitrile (10 mmol) were added until the suspension almost cleared up, then the TEBAC (150 mg) was added in the mixture as phase transfer catalyst, the mixture was stirred at 100 °C for 20–24 h, cooled to room temperature, The mixture was filtered, washed with EtOH, the solids **2f–j** formed were recrystallized from DMF and water. Their physical appearance, melting point and spectroscopic data were in agreement with published data. 

### 3.3. The Structure of Reagents R^2^-X (Figure 5) and General Procedure for the Synthesis of 1-(2-Bromoethyl)-3,7-dimethyl-1H– purine-2,6(3H,7H)-diones

Anhydrous cesium carbonate (50 mmol) and theobromine (25 mmol) were suspended in DMF (100 mL), after the mixture was stirred at room temperature for 10 min, 1,2-dibromoethane (250 mmol) was added. After completion of the reaction as monitored by TLC, the mixture was poured into water, extracted with ethyl acetate, the organic layer was washed with water twice, and dried with anhydrous sodium sulfate. The combined organic layer was evaporated to obtain a residue which was purified by column chromatography. Its physical appearance and spectroscopic data were in agreement with published data.

**Figure 5:**
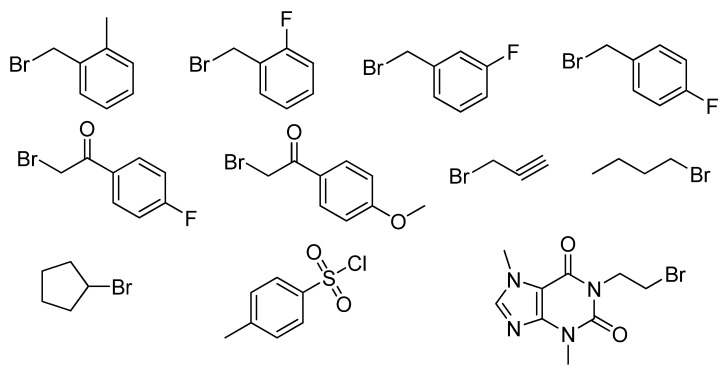
The chemical structure of reagents R^2^-X.

### 3.4. Syntheses of Compounds *3a–s*

The corresponding compounds **2 **(1 mmol) and anhydrous potassium carbonate were stirred at 60 °C (for **3f–q**, room temperature) in DMF (5 mL), then R^2^-X (1.1 mmol) was added. After completion of the reaction monitored by TLC, the mixture was poured into water (25 mL). It was extracted with dichloromethane (for **3f–q**, ethyl acetate was used). The combined organic layer was washed with water, dried with Na_2_SO_4_, evaporated to obtain a residue, and purified by column chromatography. 

*7-Amino-3-(2-(3,7-dimethyl-2,6-dioxo-2,3,6,7-tetrahydro-1H-purin-1-yl)ethyl)-2,4-dioxo-5-phenyl-1,2,3,4-tetrahydropyrido[2,3-d]pyrimidine-6-carbonitrile* (**3a**) White solid; Yield: 40.1%; ^1^H-NMR (DMSO-d_6_) δ 11.11 (s, 1H), 8.00(s, 1H), 7.65 (s, 2H), 7.41 (d, *J* = 2.4 Hz, 2H), 7.26 (t, *J* = 2.8 Hz, 1H), 7.15 (t, *J* = 3.2 Hz, 2H), 4.47 (s, 2H), 4.26 (s, 2H), 3.79 (s, 3H), 3.35 (s, 3H); ^13^C-NMR (DMSO-d_6_): δ 160.78, 159.91, 158.91, 155.46, 155.00, 154.56, 150.99, 150.54, 150.19, 148.17, 142.84, 136.65, 128.23, 127.65, 127.37, 115.24, 106.37, 98.89, 98.22, 88.61, 87.80, 33.08, 29.34; HRMS: calcd. for C_23_H_19_N_9_O_4_^+^ [M+Na^+^]: 508.1458, found: 508.1486.

7*-Amino-5-(4-chlorophenyl)-3-(2-(3,7-dimethyl-2,6-dioxo-2,3,6,7-tetrahydro-1H-purin-1-yl)ethyl)-2,4-dioxo-1,2,3,4-tetrahydropyrido[2,3-d]pyrimidine-6-carbonitrile* (**3b**) White solid; Yield: 57.3%; ^1^H-NMR (DMSO-d_6_) δ 11.17 (s, 1H), 8.00 (s, 1H), 7.69 (s, 2H), 7.49 (d, *J* = 8.8 Hz, 2H), 7.19 (d, *J* = 8.8 Hz, 2H), 4.46 (d, *J* = 3.2 Hz, 2H), 4.26 (d, *J* = 3.2 Hz, 2H), 3.79 (s, 3H), 3.35 (s, 3H); ^13^C-NMR (DMSO-d_6_): δ 159.83, 158.82, 157.56, 154.99 154.55, 150.98, 150.49, 148.18, 142.84, 135.53, 133.06, 129.41, 127.78, 114.93, 106.37, 98.84, 87.62, 67.19, 60.70, 59.72, 33.08, 29.25, 19.93; HRMS: calcd. for C_23_H_18_ClN_9_O_4_^+^ [M+Na^+^]: 542.1068, 544.1038, found: 542.1012, 544.1064.

*7-Amino-5-(3,4-dimethoxyphenyl)-3-(2-(3,7-dimethyl-2,6-dioxo-2,3,6,7-tetrahydro-1H-purin-1-yl)-ethyl)-2,4-dioxo-1,2,3,4-tetrahydropyrido[2,3-d]pyrimidine-6-carbonitrile* (**3c**) White solid; Yield: 32.2%; ^1^H-NMR (DMSO-d_6_) δ 11.09 (s, 1H), 8.00 (s, 1H), 7.58 (s, 2H), 6.99 (d, *J* = 8.4 Hz, 1H), 6.76 (d, *J* = 2 Hz,1H), 6.71 (dd, *J* = 8 Hz, *J* = 2 Hz, 1H), 4.46 (m, 2H), 4.25 (s, 2H), 3.81 (s, 3H), 3.79 (s, 3H), 3.72 (s, 3H), 3.35 (s, 3H); ^13^C-NMR (DMSO-d_6_): δ 159.87, 158.73, 155.00, 154.56, 151.00, 150.53, 148.93, 148.71, 147.82, 142.82, 128.73, 120.18, 115.34, 111.79, 110.88, 106.38, 99.03, 88.07, 67.19, 67.15, 60.70, 55.45, 33.07, 29.23, 19.92; HRMS: calcd. for C_25_H_23_N_9_O_6_^+^ [M+Na^+^]: 568.1669, found: 568.1703.

*7-Amino-3-(2-(3,7-dimethyl-2,6-dioxo-2,3,6,7-tetrahydro-1H-purin-1-yl)ethyl)-5-(4-hydroxy-3-methoxyphenyl)-2,4-dioxo-1,2,3,4-tetrahydropyrido[2,3-d]pyrimidine-6-carbonitrile* (**3d**) White solid; Yield: 30.5%; ^1^H-NMR (DMSO-d_6_) δ 11.07 (s, 1H), 9.25 (s, 1H), 7.53 (s, 2H), 6.80 (d, *J* = 8 Hz, 1H), 6.73 (d, *J* = 1.6 Hz, 1H), 6.58 (dd, *J* = 7.6 Hz, *J* = 1.2 Hz, 1H), 4.46 (d, *J* = 19.6 Hz, 2H), 4.25 (s, 2H), 3.79 (s, 3H), 3.73 (s, 3H), 3.35 (s, 3H); ^13^C-NMR (DMSO-d_6_): δ 159.88, 159.05, 158.75, 155.02(2C), 150.98, 150.54, 148.18(2C), 146.95, 146.76, 142.82, 127.23, 120.61, 114.83(2C), 112.32, 106.37, 99.03, 88.11, 55.67(2C), 33.07, 29.33; HRMS: calcd. for C_24_H_21_N_9_O_6_^+^ [M+Na^+^]: 554.1512, found: 554.1580.

*7-Amino-5-(4-bromophenyl)-3-(2-(3,7-dimethyl-2,6-dioxo-2,3,6,7-tetrahydro-1H-purin-1-yl)ethyl)-2,4-dioxo-1,2,3,4-tetrahydropyrido[2,3-d]pyrimidine-6-carbonitrile* (**3e**) White solid; Yield: 27.7%; ^1^H-NMR (DMSO-d_6_) δ 11.18 (s, 1H), 8.00 (s, 1H), 7.64 (s, 2H), 7.63 (d, *J* = 8.4 Hz, 1H), 7.13 (d, *J* = 8.4 Hz, 1H), 4,46 (s, 2H), 4.25 (s, 2H), 3.79 (s, 3H), 3.36 (s, 3H); ^13^C-NMR (DMSO-d_6_): δ 159.83, 158.83, 157.57, 155.00, 154.56, 150.99, 150.50, 148.19, 142.86, 135.94, 130.76, 129.57, 121.72, 114.93, 106.37, 98.79, 87.53, 59.72(2C), 33.09, 29.34, 20.73, 14.05; HRMS: calcd. for C_23_H_18_BrN_9_O_4_^+^ [M-H^+^]: 562.0587, 564.0566, found: 562.0511, 564.0612.

*7-Amino-3-(2-(3,7-dimethyl-2,6-dioxo-2,3,6,7-tetrahydro-1H-purin-1-yl)ethyl)-1-methyl-2,4-dioxo-5-phenyl-1,2,3,4-tetrahydropyrido[2,3-d]pyrimidine-6-carbonitrile* (**3f**) White solid; Yield: 57.8%; ^1^H-NMR (DMSO-d_6_) δ 8.08 (s, 1H), 7.89 (s, 2H), 7.34 (t, *J* = 7.6 Hz, 1H), 7.25 (t, *J* = 7.6 Hz, 2H), 6.83 (d, *J* = 7.2 Hz, 2H), 4.06 (m, 4H).3.76 (s, 3H), 3.46 (s, 3H), 3.30 (s, 3H); ^13^C-NMR (DMSO-d_6_): δ 160.75, 159.89, 158.84, 155.09(2C), 154.15, 151.51, 151.26(2C), 148.81, 143.41, 136.90, 128.49, 127.96, 127.30, 115.68, 106.96, 98.56(2C), 88.90(2C), 33.52, 29.96, 29.74; HRMS: calcd. for C_24_H_21_N_9_O_4_^+^ [M+Na^+^]: 522.1614, found: 522.1642.

*7-Amino-3-(2-(3,7-dimethyl-2,6-dioxo-2,3,6,7-tetrahydro-1H-purin-1-yl)ethyl)-5-(4-hydroxy-3-methoxyphenyl)-1-methyl-2,4-dioxo-1,2,3,4-tetrahydropyrido[2,3-d]pyrimidine-6-carbonitrile* (**3g**) White solid; Yield: 52.1%; ^1^H-NMR (DMSO-d_6_) δ 9.21 (s, 1H), 8.04 (s, 1H), 7.83 (s, 2H), 6.73 (s, 1H), 6.62 (d, *J* = 8.4 Hz, 1H), 6.20 (dd, *J* = 8.4 Hz, *J* = 1.6 Hz, 1H), 4.18 (m, 2H), 3.98 (m, 2H), 3.77 (s, 3H), 3.68 (s, 3H), 3.43 (s, 3H), 3.31(s, 3H); ^13^C-NMR (DMSO-d_6_): δ 160.77, 160.21, 159.08, 155.08, 154.65, 152.05(2C), 148.79, 147.30(2C), 143.96, 127.96, 121.33, 115.10(2C), 112.95, 107.05, 98.81, 88.78, 79.64, 56.08(2C), 33.53, 29.95, 29.77; HRMS: calcd. for C_25_H_23_N_9_O_6_^+^ [M+Na^+^]: 568.1669, found: 568.1605.

*7-Amino-5-(3,4-dimethoxyphenyl)-3-(2-(3,7-dimethyl-2,6-dioxo-2,3,6,7-tetrahydro-1H-purin-1-yl) ethyl)-1-methyl-2,4-dioxo-1,2,3,4-tetrahydropyrido[2,3-d]pyrimidine-6-carbonitrile* (**3h**) White solid; Yield: 49.5%; ^1^H-NMR (DMSO-d_6_) δ 8.06 (s, 1H), 7.87 (s, 2H), 7.77 (s, 1H), 6.76 (d, *J* = 8.4 Hz, 1H), 6.35 (dd, *J* = 1.6 Hz, *J* = 8 Hz, 1H), 4.15 (m, 2H), 4.22 (m, 2H), 3.79 (s, 3H), 3.78 (s, 3H), 3.67 (s, 3H), 3.43 (s, 3H), 3.31 (s, 3H); ^13^C-NMR (DMSO-d_6_): δ160.43, 159.84, 158.55, 154.87, 154.13, 151.59, 151.35, 148.81, 148.38, 143.56, 129.52, 120.22, 115.67(2C), 112.13, 106.99, 98.87, 88.40, 79.22, 55.98, 55.86, 33.55, 29.96, 29.78, 21.22, 14.55; HRMS: calcd. for C_26_H_25_N_9_O_6_^+^ [M+Na^+^]: 582.1825, found: 582.1877.

*7-Amino-3-(2-(3,7-dimethyl-2,6-dioxo-2,3,6,7-tetrahydro-1H-purin-1-yl)ethyl)-1-methyl-2,4-dioxo-5-(thiophen-2-yl)-1,2,3,4-tetrahydropyrido[2,3-d]pyrimidine-6-carbonitrile* (**3i**) White solid; Yield: 60.3%; ^1^H-NMR (DMSO-d_6_) δ 8.04 (s, 1H), 7.96 (s, 2H), 7.59 (d, *J* = 4.8 Hz, 1H), 7.01 (t, *J* = 3.2 Hz, 1H), 6.81 (d, *J* = 2.4 Hz, 1H), 4.08 (d, *J* = 5.2 Hz, 4H), 3.77 (s, 3H), 3.44 (s, 3H), 3.31 (s, 3H); ^13^C-NMR (DMSO-d_6_): δ 161.03, 158.95, 154.12, 151.05, 149.88, 148.62, 137.76, 136.25, 130.89, 128.43, 128.02, 123.52, 116.83, 112.04, 111.80, 101.62, 90.85, 56.66, 55.89, 45.73, 30.68, 16.98; HRMS: calcd. for C_22_H_19_N_9_O_4_S^+^ [M+Na^+^]: 528.1178, found: 528.1096.

*7-Amino-5-(3,4-dimethoxyphenyl)-1-methyl-3-(2-methylbenzyl)-2,4-dioxo-1,2,3,4-tetrahydropyrido[2,3-d]pyrimidine-6-carbonitrile* (**3j**) White solid; Yield: 57.8%; ^1^H-NMR (CDCl_3_) δ 7.12 (s, 1H), 7.11 (d, *J* = 1.2 Hz, 1H), 7.06 (m, 1H), 6.96 (d, *J* = 8 Hz, 2H), 6.90 (dd, *J* = 8 Hz, *J* = 2 Hz, 1H), 6.73 (d, *J* = 2 Hz, 1H), 5.70 (s, 2H), 5.10 (s, 2H), 3.92 (s, 3H), 3.80 (s, 3H), 3.64 (s, 3H);^ 13^C-NMR (DMSO-d_6_): δ 160.16(2C), 158.77, 154.22, 151.05, 149.88, 148.62, 135.76, 134.44, 130.30, 128.23, 127.02, 125.84, 125.59, 120.20, 115.53, 111.24, 110.70, 100.42, 90.35, 55.89, 55.75, 42.23, 30.27, 19.26; HRMS: calcd. for C_25_H_23_N_5_O_4_^+^ [M+Na^+^]: 480.1648, found: 480.1604.

*7-Amino-5-(3-methoxy-4-(2-methylbenzyloxy)phenyl)-1-methyl-3-(2-methylbenzyl)-2,4-dioxo-1,2,3,4-tetrahydropyrido[2,3-d]pyrimidine-6-carbonitrile* (**3k**) White solid; Yield: 28.8%; ^1^H-NMR (CDCl_3_) δ 7.46 (d, *J* = 7.2 Hz, 1H), 7.26–7.21 (m, 3H), 7.13 (t, *J* = 2.8 Hz, 2H), 7.10–7.07 (m, 1H), 7.02 (d, *J* = 8.8 Hz, 1H), 6.97 (d, *J* = 7.6 Hz, 1H), 6.88 (dd, *J* = 8 Hz, *J* = 2Hz, 1H), 6.78 (d, *J* = 1.6 Hz, 1H), 5.71 (s, 2H), 5.15(s, 2H), 5.13 (s, 2H), 3.80 (s, 3H), 3.66 (s, 3H), 2.41 (s, 3H), 2.39 (s, 3H); ^13^C-NMR (DMSO-d_6_): δ 160.88, 159.84, 158.73, 154.40, 151.28(2C), 148.71, 148.59, 137.28, 135.40, 135.29, 135.26, 130.56, 130.26, 129.97, 129.33, 128.63, 126.91, 126.26, 125.04, 120.46, 116.11, 112.76, 112.47, 99.29, 89.36, 68.96, 55.98, 42.23, 30.24, 19.13, 18.94; HRMS: calcd. for C_32_H_29_N_5_O_4_^+^ [M-H^+^]: 546.2141, found: 546.2157.

*7-Amino-1-methyl-3-(2-methylbenzyl)-2,4-dioxo-5-phenyl-1,2,3,4-tetrahydropyrido[2,3-d]pyrimidine-6-carbonitrile* (**3l**) White solid; Yield: 65.8%; ^1^H-NMR (CDCl_3_) δ 7.48 (t, *J* = 3.2 Hz, 3H), 7.28 (d, *J* = 3.6 Hz, 2H), 7.11 (d, *J* = 3.6 Hz, 2H), 7.07 (m, 1H), 6.90 (d, *J* = 7.6 Hz, 1H), 5.71 (s, 2H), 5.08 (s, 2H), 3.65 (s, 3H), 2.35 (s, 3H); ^13^C-NMR (DMSO-d_6_): δ 160.49, 159.92, 158.82, 154.24, 151.32, 150.06, 148.77, 136.18, 135.63, 134.28, 130.26, 129.19, 128.32, 127.07, 126.99, 125.91, 125.35, 115.19, 100.56, 90.60, 42.20, 30.26, 19.21; HRMS: calcd. for C_23_H_19_N_5_O_2_^+^ [M+H^+^]: 398.1617, found: 398.1685.

*7-Amino-3-(2-fluorobenzyl)-1-methyl-2,4-dioxo-5-phenyl-1,2,3,4-tetrahydropyrido[2,3-d]pyrimidine-6-carbonitrile* (**3m**) White solid; Yield: 60.2%; ^1^H-NMR (CDCl_3_) δ 7.51 (t, *J* = 2.8 Hz, 3H), 7.38 (dd, *J* = 8.4 Hz, *J* = 5.2 Hz, 2H), 7.26 (s, 2H), 6.93 (t, *J* = 8.8 Hz, 2H), 5.68 (s, 2H), 5.03 (s, 2H), 3.62 (s, 3H), 2.18 (s, 3H); ^13^C-NMR (DMSO-d_6_): δ 160.49, 159.94, 159.87, 158.70, 154.12, 150.80, 136.14, 131.03, 130.98, 129.25, 128.95, 128.92, 128.35, 127.10, 123.98(2C), 115.42, 115.14, 100.37, 90.26, 43.84, 38.67, 30.21; HRMS: calcd. for C_22_H_16_FN_5_O_2_^+^ [M-H^+^]: 400.1210, found: 400.1248.

*7-Amino-3-(3-fluorobenzyl)-1-methyl-2,4-dioxo-5-phenyl-1,2,3,4-tetrahydropyrido[2,3-d]pyrimidine-6-carbonitrile* (**3n**) White solid; Yield: 66.7%; ^1^H-NMR (CDCl_3_) δ 7.51 (t, *J* = 3.2 Hz, 3H), 7.28–7.19 (m, 3H), 7.14 (d, *J* = 7.6 Hz, 1H), 7.05 (d, *J* = 10 Hz, 1H), 6.94–6.89 (m, 1H), 5.70 (s, 2H), 5.05 (s, 2H), 3.63 (s, 3H);^ 13^C-NMR (DMSO*-*d_6_): δ 163.94, 161.49, 160.47, 159.95, 158.68, 154.07, 150.98, 139.06, 136.21, 129.82, 129.24, 128.35, 127.11, 124.43, 115.62, 115.13, 115.38, 114.41, 100.39, 90.31, 44.09, 30.22; HRMS: calcd. for C_22_H_16_FN_5_O_2_^+^ [M-H^+^]: 400.1210, found: 400.1296.

*7-Amino-3-(2-(4-fluorophenyl)-2-oxoethyl)-1-methyl-2,4-dioxo-5-phenyl-1,2,3,4-tetrahydropyrido[2,3-d]pyrimidine-6-carbonitrile* (**3o**) Yellow solid; Yield: 48.0%; ^1^H-NMR (CDCl_3_) δ 7.95 (dd, *J* = 8.8, *J* = 5.2 Hz, 2H), 7.46 (t, *J* = 3.6Hz, 3H), 7.26 (d, *J* = 9.2Hz, 2H), 7.12 (t, *J* = 8.6 Hz, 2H), 5.74 (s, 2H), 5.32 (s, 2H), 3.66 (s, 3H); ^13^C-NMR (DMSO-d_6_): δ 191.68, 166.53, 160.90, 160.12, 158.37, 154.23, 151.00, 137.43, 131.60, 131.53, 128.64, 128.23(2C), 127.68(3C), 116.55, 116.41, 115.65, 98.69, 89.53, 47.82, 30.20; HRMS: calcd. for C_23_H_16_FN_5_O_3_^+^ [M-H^+^]: 428.1159, found: 428.1105.

*7-Amino-3-(2-(4-methoxyphenyl)-2-oxoethyl)-1-methyl-2,4-dioxo-5-phenyl-1,2,3,4-tetrahydropyrido[2,3-d]pyrimidine-6-carbonitrile* (**3p**) Yellow solid; Yield: 45.2%; ^1^H-NMR (CDCl_3_) δ 7.90 (d, *J* = 8.8 Hz, 2H), 7.46 (t, *J* = 4 Hz, 3H), 7.27 (d, *J* = 9.2 Hz, 2H), 6.91 (d, *J* = 8.8 Hz, 2H), 5.73 (s, 2H), 5.32 (s, 2H), 3.86 (s, 3H), 3.67 (s, 3H); ^13^C-NMR (DMSO-d_6_): δ 191.11, 164.13, 160.88, 160.11, 158.40, 154.22, 151.03, 137.46, 130.77(2C), 128.63, 128.23(2C), 127.69(2C), 115.68, 114.57(2C), 98.72, 89.50, 56.07(2C), 47.56, 30.18; HRMS: calcd. for C_24_H_19_N_5_O_4_^+^ [M-H^+^]: 440.1359, found: 440.1375.

*7-Amino-1-methyl-2,4-dioxo-5-phenyl-3-(prop-2-ynyl)-1,2,3,4-tetrahydropyrido[2,3-d]pyrimidine-6-carbonitrile* (**3q**) White solid; Yield: 65.7%; ^1^H-NMR (DMSO-d_6_) δ 7.95 (s, 2H), 7.44 (t, *J* = 3 Hz, 3H), 7.25–7.23 (m, 2H), 7.44 (d, *J* = 1.6 Hz, 2H), 3.52 (s, 3H), 3.07 (s, 1H); ^13^C-NMR (DMSO*-*d_6_): δ 160.82, 160.13, 157.85, 154.17, 150.50, 137.52, 128.67, 128.28(2C), 127.69(2C), 115.66, 98.86, 89.41, 79.67, 73.45, 30.67, 30.18; HRMS: calcd. for C_18_H_13_N_5_O_2_^+^ [M-H^+^]: 330.0991, found: 330.0916.

*7-Amino-3-cyclopentyl-1-methyl-2,4-dioxo-5-phenyl-1,2,3,4-tetrahydropyrido[2,3-d]pyrimidine-6-carbonitrile* (**3r**) White solid; Yield: 48.3%; ^1^H-NMR (CDCl_3_) δ 7.51–7.49 (m, 3H), 7.25 (t, *J* = 4 Hz, 2H), 5.66 (s, 2H), 5.26–5.21 (m, 1H), 3.61 (s, 3H), 2.05–1.99 (m, 2H), 1.90–1.85 (m, 2H), 1.79–1.71 (m, 2H), 1.52–1.48 (m, 2H); ^13^C-NMR (DMSO*-*d_6_): δ 160.73, 160.02, 159.12, 154.08, 150.70, 137.96, 128.48, 128.22(2C), 127.64(2C), 115.80, 99.37, 89.10, 52.83, 29.88, 28.37(2C), 25.66(2C); HRMS: calcd. for C_20_H_19_N_5_O_2_^+ ^[M-H^+^]: 360.1460, found: 360.1436.

*7-Amino-3-butyl-1-methyl-2,4-dioxo-5-phenyl-1,2,3,4-tetrahydropyrido[2,3-d]pyrimidine-6-carbo-nitrile* (**3s**) White solid; Yield: 51.9%; ^1^H-NMR (CDCl_3_) δ 7.50 (t, *J* = 3.6 Hz, 3H), 7.25 (d, *J* = 2.8 Hz, 2H), 5.67 (s, 2H), 3.86 (t, *J* = 7.8 Hz, 2H), 3.64 (s, 3H), 1.56–1.48 (m, 2H), 1.31–1.25 (m, 2H), 0.89–0.86 (m, 3H); ^13^C-NMR (DMSO*-*d_6_): δ 160.23, 159.80, 158.64, 154.01, 151.00, 136.37, 129.14, 128.31(2C), 127.00(2C), 115.26, 100.59, 90.06, 41.69, 30.10, 29.74, 20.15, 13.75; HRMS: calcd. for C_19_H_19_N_5_O_2_^+^ [M-H^+^] m/z 348.1460, found: 348.1488.

### 3.5. Synthesis of Compounds *4a–d*

Compounds **2g** or **2j** (1 mmol) and anhydrous potassium carbonate were stirred at room temperature in DMF (5 mL). Reagents 1-(bromomethyl)-4-fluorobenzene or 2-bromo-1-(4-methoxyphenyl) ethanone (2.1 mmol) were added. After monitoring the completion of the reaction by TLC, the mixture was poured into water (25 mL). It was extracted with EA. The combined organic layer was washed with water, dried with Na_2_SO_4_, evaporated to obtain a residue, that was purified by column chromatography.

*7-Amino-3-(4-fluorobenzyl)-5-(4-(4-fluorobenzyloxy)-3-methoxyphenyl)-1-methyl-2,4-dioxo-1,2,3,4-tetrahydropyrido[2,3-d]pyrimidine-6-carbonitrile* (**4a**) White solid; Yield: 27.2%; ^1^H-NMR (CDCl_3_) δ 7.47–7.40 (m, 4H), 7.08 (t, *J* = 8.6 Hz, 2H), 7.00 (d, *J* = 8.4 Hz, 1H), 6.94 (t, *J* = 8.8 Hz, 2H), 6.84 (dd, *J* = 8.4 Hz, *J* = 2 Hz, 1H), 7.75 (d, *J* = 2 Hz, 1H), 5.67 (s, 2H), 5.15 (s, 2H), 5.05 (s, 2H), 3.83 (s, 3H), 3.62 (s, 3H); ^13^C-NMR (DMSO-d_6_): δ 163.04, 162.44, 160.62, 160.33, 160.03, 159.29, 158.17, 153.77, 150.78, 148.18, 147.93, 133.38, 133.24, 133.21, 130.25, 130.17, 129.64, 119.97, 115.56, 115.33, 115.12, 114.99, 114.78, 112.36, 112.06, 98.74, 88.91, 69.13, 55.51, 29.69; HRMS: calcd. for C_30_H_23_F_2_N_5_O_4_^+^ [M+H^+^]: 556.1796, found: 556.1856.

*7-Amino-3-(4-fluorobenzyl)-5-(4-(4-fluorobenzyloxy)phenyl)-1-methyl-2,4-dioxo-1,2,3,4-tetrahydropyrido-[2,3-d]pyrimidine-6-carbonitrile* (**4b**) White solid; Yield: 24.6%; ^1^H-NMR (CDCl_3_) δ 7.55 (t, *J* = 7.6 Hz, 1H), 7.36–7.00 (m, 11H), 5.72 (s, 2H), 5.20 (s, 2H), 5.19 (s, 2H), 3.64 (s, 3H); ^13^C-NMR (DMSO-d_6_): δ 161.47, 160.30, 159.71, 158.82, 154.20, 150.79, 129.95, 129.88, 129.82, 129.04, 128.95, 128.43, 124.34, 123.98, 123.96, 123.88, 123.78, 123.64, 123.54, 115.48, 115.34, 115.32, 114.44, 100.40, 90.39, 63.64, 63.61, 38.71, 30.24; HRMS: calcd. for C_29_H_21_F_2_N_5_O_3_^+^ [M+H^+^]: 526.1691, found: 526.1675.

*7-Amino-5-(3-methoxy-4-(2-(4-methoxyphenyl)-2-oxoethoxy)phenyl)-3-(2-(4-methoxyphenyl)-2-oxo-ethyl)-1-methyl-2,4-dioxo-1,2,3,4-tetrahydropyrido[2,3-d]pyrimidine-6-carbonitrile* (**4c**) White solid; Yield: 28.1%; ^1^H-NMR (DMSO-d_6_) δ 8.01 (dd, *J* = 8.8 Hz, *J* = 6.8 Hz, 4H), 7.07 (dd, *J* = 8.4 Hz, *J* = 5.2 Hz, 4H), 6.90–6.85 (m, 2H), 6.73(dd, *J* = 8.8 Hz, *J* = 2 Hz, 1H), 5.50 (s, 2H), 5.23 (s, 2H), 3.85 (s, 6H), 3.74 (s, 3H), 3.53 (s, 3H); ^13^C-NMR (DMSO-d_6_): δ 193.22, 191.18, 164.14, 164.02, 160.90, 159.84, 158.36, 154.22, 151.04, 148.50, 148.10, 130.77(4C), 130.01, 127.77, 127.71, 120.37, 115.97, 114.59(2C), 114.50(2C), 112.97, 112.64, 98.88, 89.67, 70.87, 56.13, 56.07, 47.61, 30.18; HRMS: calcd. for C_34_H_29_N_5_O_8_^+ ^[M+Na^+^]: 658.1914, found: 658.1896.

*7-Amino-5-(4-(2-(4-methoxyphenyl)-2-oxoethoxy)phenyl)-3-(2-(4-methoxyphenyl)-2-oxoethyl)-1-methyl-2,4-dioxo-1,2,3,4-tetrahydropyrido[2,3-d]pyrimidine-6-carbonitrile* (**4d**) White solid; Yield: 21.7%; ^1^H-NMR (DMSO-d_6_) δ 8.01 (t, *J* = 9.2 Hz, 4H), 7.17 (d, *J* = 8.8 Hz, 2H), 7.10–7.03 (m, 4H), 6.96 (d, *J* = 8.8 Hz, 2H), 5.53 (s, 2H), 5.22 (s, 2H), 3.86 (s, 6H), 3.53 (s, 3H); ^13^C-NMR (DMSO-d_6_): δ 193.14, 191.15, 164.13, 164.03, 160.90, 160.50, 159.94, 158.58, 158.45, 158.14, 154.26, 151.02, 130.79(2C), 130.73(2C), 129.63, 129.46, 129.39, 127.72, 127.68, 114.98, 114.54(2C), 114.30(2C), 98.82, 89.66, 70.33, 56.07(2C), 47.58, 30.19; HRMS: calcd. for C_33_H_27_N_5_O_7_^+^ [M+H^+^]:606.1989, found: 606.1971.

### 3.6. Synthesis of Compound *4e*

Compound **2g** (1 mmol) and TsCl (2.1 mmol) were dissolved in DMF (5 mL) followed by the addition of triethylamine (3 mmol). The mixture was stirred at room temperature for 24 h. The reaction mixture was poured into water and extracted with DCM. The combined organic layer were washed with water and evaporated to half of its volume. The organic layer was cooled to room temperature, and the solid filtered and washed with EtOH to obtain *4-(7-amino-6-cyano-1-methyl-2,4-dioxo-1,2,3,4-tetrahydropyrido[2,3-d]pyrimidin-5-yl)-2-methoxy phenyl-4-methylbenzenesulfonate* (**4e**) White solid; Yield: 57.0%; ^1^H-NMR (DMSO-d_6_) δ 11.24 (s, 1H), 7.89 (s, 2H), 7.62 (d, *J* = 8.4 Hz, 2H), 7.42 (d, *J* = 8 Hz, 2H), 7.21 (d, *J* = 8 Hz, 1H), 6.92 (d, *J* = 1.6 Hz, 1H), 6.84 (dd, *J* = 8.4 Hz, *J* = 1.6 Hz, 1H), 3.42 (s, 3H), 3.34 (s, 3H), 2.41 (s, 3H); ^13^C-NMR (DMSO*-*d_6_): δ 162.77, 160.74, 159.24, 158.29, 155.40, 151.15, 150.96, 145.90, 137.86, 137.74, 132.21, 130.15, 128.74, 123.57, 120.31, 115.66, 113.28, 99.70, 88.48, 56.07, 36.24, 29.12, 21.62; HRMS: calcd. for C_23_H_19_N_5_O_6_S^+^ [M+H^+^]: 494.1134, found: 494.1108.

### 3.7. Cell Proliferation Assay (MTT Assay)

Briefly, cells (2,500/well) were seeded in 96-well plates and cultured for 24 h, followed by treatment with target compounds for a further 48 h. Twenty microlitres of 5 mg/mL MTT was added per well and incubated for a further 2.5 h at 37 °C. Then the supernatant was removed, and 150 μL/well DMSO added for 15–20 min. The optical density of each well was measured at 570 nm using a SpectraMAX M5 microplate spectrophotometer (Molecular Devices, Silicon Valley, CA, USA).

### 3.8. Apoptosis Analyses

Briefly, 1.5 × 10^5^ cells were seeded per well in a six-well plate. Twenty-four hours later, cells were treated with **4a** for a further 48 h. All cells were collected, washed twice with phosphate-buffered saline (PBS) and resuspended in 100 μL binding buffer. Then cell suspensions were mixed with 5 μL annexin V-FITC and 10 μL PI, and incubated for 15 min in the dark at room temperature. After staining, 400 μL of binding buffer was added and stained cells analyzed using a flow cytometer.

## 4. Conclusions

Twenty-four novel N-3 substituted 7-aminopyrido[2,3-*d*]pyrimidin-6-carbonitrile derivatives were synthesized and evaluated as potential anticancer agents through a cell-based phenotypic screening approach. Compound **3k** was found to exhibit significant potency against human colon cancer cells SW620 with an IC_50_ value of 12.5 μM. Further structural modification led to a more potent compound **4a** with an IC_50_ value of 6.9 μM, which is slightly more potent than cisplatin against SW620 tumor cells. Preliminary SARs information suggested that lipophilic groups at N-3 position are preferred and a methoxy group at the R^4^-position plays a crucial part. Flow cytometric analyses indicated that compound **4a** acts through induction of apoptosis in human colon cancer SW620 cells. The underlying mechanism of action of compound **3k** and **4a** will further examined in our lab. 
